# The influence of dairy consumption, sedentary behaviour and physical activity on bone mass in Flemish children: a cross-sectional study

**DOI:** 10.1186/s12889-015-2077-7

**Published:** 2015-07-28

**Authors:** Isabelle Sioen, Nathalie Michels, Carolien Polfliet, Stephanie De Smet, Sara D’Haese, Inge Roggen, Jean Deschepper, Stefan Goemaere, Jara Valtueña, Stefaan De Henauw

**Affiliations:** Department of Public Health, Faculty of Medicine and Health Sciences, Ghent University, 4K3, De Pintelaan 185, B-9000 Ghent, Belgium; FWO, Research Foundation Flanders, Egmontstraat 5, 1000 Brussels, Belgium; Department of Movement and Sport Sciences, Faculty of Medicine and Health Sciences, Ghent University, Ghent, Belgium; Department of Pediatrics, Universitair Ziekenhuis Brussel, Vrije Universiteit Brussel, Brussels, Belgium; Department of Pediatrics, Ghent University Hospital, Ghent, Belgium; Department of Endocrinology, Faculty of Medicine and Health Sciences, Ghent University, Ghent, Belgium; Unit for Osteoporosis and Metabolic Bone Diseases, Ghent University Hospital, Ghent, Belgium; ImFine Research Group, Department of Health and Human Performance, Faculty of Physical Activity and Sport Sciences, Universidad Politécnica de Madrid, Madrid, Spain; Department of Health Sciences, Vesalius, University College Ghent, Ghent, Belgium

**Keywords:** Bone mineral content (BMC), Areal bone mineral density (aBMD), Children, Dairy consumption, Dual-energy X-ray absorptiometry (DXA), Physical activity, Sedentary behaviour

## Abstract

**Background:**

This cross-sectional study aimed to look for an association in young children between whole body bone mineral content (BMC) and areal bone mineral density (aBMD) and dairy consumption as well as sedentary behaviour (SB) and physical activity (PA). Moreover, we investigated whether there was an interaction effect between dairy consumption and SB or PA on BMC and aBMD.

**Methods:**

Healthy children (6-12 years) were recruited from primary schools. Body composition and whole body bone mass were measured with dual-energy X-ray absorptiometry (DXA), dairy consumption was assessed with a food frequency questionnaire (FFQ) and PA and SB with an accelerometer. In total, 272 children underwent a DXA scan. Complete FFQ data were available for 264 children and 210 children had matching data from accelerometry recordings. Regression analyses were used to study the associations between (1) BMC and aBMD and (2) dairy consumption, SB and PA, adjusting for age, gender, pubertal stage, height and body composition.

**Results:**

Dairy consumption was positively associated with whole body BMC and aBMD (absolute value as well as z-score), after correction for relevant confounders. SB was negatively associated with aBMD z-score and light PA was positively associated with both BMC and aBMD z-score. No gender differences were found. Moreover, an interaction effect between vigorous PA (VPA) and dairy consumption on aBMD (z-score) and BMC z-score was found, indicating that children with both high VPA and high dairy consumption had higher values for BMC and aBMD of the whole body minus the head.

**Conclusion:**

Already at young age, PA and dairy consumption positively influence whole body bone mass assessed by DXA. Moreover, this study indicates clearly that SB is negatively associated with whole body bone density. Promoting regular PA and sufficient dairy consumption in young children and limiting SB can be expected to positively influence their bone mass accumulation, which can help in the prevention of osteoporosis later in life.

## Background

Osteoporosis is a well-known major public health problem. It occurs most commonly in the elderly, but evidence is accumulating that predisposing factors arise in childhood and adolescence [1, 2]. The optimization of peak bone mass in early adulthood is a major preventive measure of osteoporosis later in life. Peak bone mass is defined as the highest level of bone mass (BM) achieved during life. BM is a more generalized term describing the amount of mineral. Bone mineral content (BMC; g) quantifies the amount of mineral measured within a sub region. Areal bone mineral density (aBMD; g/cm^2^) is determined by dividing the BMC (g) by the surface area (cm^2^) of the region of interest as measured by dual-energy X-ray absorptiometry (DXA) [3, 4]. Childhood and adolescence are important life stages for bone development since peak bone mass is reached at the end of adolescence.

Lifestyle-related factors including physical activity (more specifically weight-bearing exercise) and dietary habits (more specifically dairy consumption and vitamin D intake) contribute up to 20 % in the variation in peak bone mass [5]. An adequate amount of dietary calcium, of which dairy products are a rich source, is important for reaching a normal bone mass at completion of pubertal growth. Moreover, dairy products also contain other components that help to increase bone mass, e.g. high biological value proteins and phosphorus as well as other minerals and vitamins [6, 7]. Next, physical activity is well known to have a positive effect on bone development during childhood. In this relation, muscle mass plays an important role. This is explained by the mechanostat theory stating that changes in the habitual environmental loading will lead to changes in the skeletal mass. As a result, skeletal overloading will lead to an increase in bone mass [8].

Furthermore, the combined effect or interaction between physical activity and dairy consumption might be more efficacious for maximal bone mass accumulation than just exercise or dairy consumption. Some interactions between calcium intake and physical activity on BM have been found in European adolescents [9, 10], but it is still not clear whether this interaction effect applies to young children [11]. Moreover, results from some observational and intervention studies have been inconsistent and this is mainly attributed to the different gender and age-stage studies, the effect of hormonal changes, reliance on recalled data, and the limitations of current dietary assessment methods.

In contrast to the positive effects of physical activity on BM, more and more studies also indicate the fact that sedentary behaviour is a determinant of low BM [12–16]. However, no studies are available yet on the influence of sedentary behaviour on bone mass in young children from 6 years onwards.

So far, the number of studies investigating simultaneously the effect of sedentary behaviour, physical activity and dairy consumption on bone health in young children (below the age of 12 years) is limited indicating the need for more research in this field. Therefore, this cross-sectional study aimed to look for an association in young children between BMC and aBMD on the one hand and dairy consumption, sedentary behaviour and physical activity on the other hand. Moreover, an eventual interaction between dairy consumption and physical activity or sedentary behaviour on BMC and aBMD was investigated. In this study sedentary behaviour as well as physical activity were measured with an objective method, i.e. accelerometry, instead of using questionnaires, being an important strength of this study.

## Methods

### Study population

Participants were Belgian children recruited by random cluster design (all children from 12 schools of the selected commune named Aalter) for the longitudinal ChiBS (Children’s Body composition and Stress) study (2010-2012). The study aimed at examining the relation of stress with lifestyle and body composition [17]. At the start of the ChiBS study (2010), a sample size calculation was performed and is described by Michels et al. [17]. In total, 523 children participated in the baseline measurement of the study. At the final follow-up measurement of the ChiBS study in 2012, a DXA measurement was added as an extra module to the ChiBS study, leading to the dataset used in this paper. No new sample size calculation was done in the framework of this follow-up measurement.

For this final follow-up measurement (February - April 2012), the ChiBS children were telephonically invited to make an appointment for the follow-up measurements concerning body composition (fat, lean and bone mass) and lifestyle. An individual appointment was made in the local sports facilities of the commune of Aalter. In total, 330 children participated in the follow-up measurement (193 children were lost between 2010 and 2012) of which 272 children underwent a DXA measurement, as this was an optional module. Complete dairy consumption data of 264 children were available. Only 210 children had matching data from accelerometry, since a limited number of accelerometers were available during the survey.

The study was conducted according to the guidelines laid down in the Declaration of Helsinki 1964 (revision of Edinburgh 2000) and was approved by the Ethics Committee of the Ghent University Hospital. Parents signed an informed consent.

### Bone, lean and fat mass measurements by DXA

Children’s height (cm) was measured on bare feet using a stadiometer (SECA 225). DXA, more specifically the Hologic (Discovery-W apparatus using software version 12.8.0), was used to measure osseous and soft tissue. The device was calibrated daily using a lumbar spine phantom as recommended by the manufacturer. Children having a plaster were excluded (this was the case in one child), as well as children having an internal defibrillator, a pacemaker or osteo-synthetic material (this situation was not encountered). BMC (g) and aBMD (g/cm^2^) measurements were obtained from whole body as well as whole body minus the head measurements. Only the BMC and aBMD of the whole body minus the head were used in this study as this is – beside the lumbar spine - the most accurate and reproducible skeletal site for DXA assessment in children [18]. Additionally, for each individual a BMC and aBMD z-score was calculated according to gender, age and height based on a Belgian reference population (data not published yet). This reference population consisted of 556 healthy Caucasian children (of which 311 girls) aged 5 to 19 years, recruited in six different geographical areas in Belgium (Keerbergen, Namur, Jette, Alsemberg, Ghent and Aalter) between April 2010 and April 2012. In the different provincial areas of Belgium, several schools were invited to participate; those who replied within the study period and were able to accommodate the equipment, were chosen to be included. The participating schools were not only from different geographical areas, but recruited children from different socio-economic status, as based on the income statistics of their respective city. In six of these cities (Keerbergen, Namur, Jette, Alsemberg and Ghent), schools were selected on their willingness to participate. All children and parents in the participating schools received an information letter with detailed explanation on the study, an informed consent and a detailed questionnaire. Participation was on a voluntary basis. The children in Aalter were recruited in the framework of the ChiBS study.

Besides bone mass, total fat mass (kg) and lean mass (kg) were measured by DXA. Fat mass index (FMI, kg/m^2^) and fat-free mass index (FFMI, kg/m^2^) were calculated by dividing respectively the fat and lean mass by the height^2^.

### Accelerometer recordings

Daily physical activity was measured by an ActiGraph™ (GT3x (triaxial), GT1M (uniaxial) or actitrainer (uniaxial)) accelerometer which was worn on an elastic belt on the level of the right hip during waking hours for five (week and weekend) consecutive days. As uniaxial accelerometers only register velocity in vertical direction and as it was not possible to provide all children with a triaxial accelerometer, only the vertical axis counts were used for this study. The children were allowed to take it off for showering, bathing, water sports or in sports with a high risk of damaging the monitor. Activity counts were stored at a time interval (i.e. epoch) of 15 s intervals.

Only the data from children with readings of at least eight hours per day, but not exceeding 18 h per day and this for at least three days were used for analysis [19]. When periods of more than 20 min of consecutive zero counts were found, these periods were also classified as non-wearing time [20, 21]. The overall time (minutes) spent in sedentary behaviour, light, moderate and vigorous physical activity were calculated using the cut-off points of Evenson depending on the counts per minute (cpm): sedentary behaviour (0-100 cpm), light (100-2295 cpm), moderate (2296-4011 cpm) and vigorous (≥4012 cpm) [22, 23]. Time spent in moderate-to-vigorous physical activity (MVPA) was calculated as the sum of both the time in moderate and vigorous activity. Next, the amount of minutes spent in sedentary behaviour as well as the amount of minutes spent in each category of physical activity was expressed as a percentage by dividing the amount of minutes per category by the total recording time in order to standardise the data for total wearing time (which was different between the children). The software Meter plus 4.2 was used to screen, score and clean the accelerometer data files.

### Dairy consumption

A parentally reported, semi-quantitative food frequency questionnaire (FFQ) was used for dietary analysis. This FFQ was based on the validated FFQ of Huybrechts et al. [24]. The central question was ‘How often did your child eat or drink the following food item during the last month and which quantity of the food items is on average consumed on a regular day?’. To report the consumption frequency, 6 possible answers could be chosen ranging from ‘never’ to ‘daily’ (never, less than once a week, one day a week, 2 to 4 days a week, 5 to 6 days a week, daily). To report the consumed quantity, 5 or 6 possible answers could be chosen, expressed in g or ml per day, depending on the food item. Examples of portion sizes were given to facilitate answering the questions. In total, 67 food items were considered in the FFQ, grouped in 16 food groups. The last food group focused on food supplements. The consumption of milk, soy milk, quark, cheese and yoghurt was summed up and is refered to as total dairy consumption (g/week).

### Pubertal development

Stage of pubertal development was assessed by a paediatrician using the Tanner score of pubic hair distribution and genital development for boys and pubic hair distribution and breast development for girls [25]. In the absence of the paediatrician on the examination day, a parentally reported questionnaire with images was used to determine Tanner stage (9 % of the study population). For the analysis, the different Tanner stages were recorded in a dichotomous variable (no signs of puberty, signs of puberty) because of the low percentage of children who had started pubertal development.

### Socioeconomic status

To describe socio-economic status, parental education level (PEL) according to the International Standard Classification of Education classification (level 0 ‘pre-primary education’ , 1 ‘primary education’ , 2 ‘lower secondary education’ , 3 ‘upper secondary education’ , 4 ‘post-secondary non-tertiary education’ , 5 ‘first stage of tertiary education’ , 6 ‘second stage of tertiary education’) was used. The highest classification among the two parents was used in the analysis. Because none of the parents were classified in group 1, and only a few in group 2, the PEL levels were recoded into three groups: ‘equal or lower than level 3’ , ‘level 4’ and ‘level 5 or 6'.

### Statistical analyses

IBM SPSS Statistics 22.0 for Windows (SPSS Inc, Chicago, IL) was used to perform the statistical analyses. The level of statistical significance was set at 5 %, also for the interaction terms. Independent samples t-test or Mann-Whitney U test (for continuous variables) and chi square test (for categorical variables) were used to explore possible differences in bone parameters, body composition, accelerometer data and dairy consumption between boys and girls. BMC or aBMD differences between PEL groups were tested using ANOVA.

Multiple linear regression analyses were used to examine the associations of accelerometer data and dairy consumption with aBMD and BMC as outcome, using both the absolute values of the bone parameters as well as the z-scores. All residuals showed a satisfactory pattern. When absolute values of aBMD and BMC were included in the regression analyses as dependent variable, we adjusted for age, gender, Tanner stage, height and body composition (FMI and FFMI). When z-scores of aBMD and BMC (standardised for age, gender and height) were included in the regression analyses as dependent variable, we adjusted only for Tanner stage and body composition (FMI and FFMI). In these regression models, FMI and FFMI were included after ln-transformation due to their skewed distribution. Age was included in the models as a dichotomous variable (below or above the median age) as age was too highly correlated with height. In each multiple regression model, the collinearity between the different independent variables was checked using the variance inflation factor.

In a first set of regression analyses, we investigated the associations of sedentary behaviour, the time spent in different categories of physical activity (light, moderate, vigorous and moderate to vigorous) and of dairy consumption on bone parameters separately. Standardised regression coefficients, p-values and eta^2^ for the independent variables are reported for each model. The eta^2^ describes the proportion out of the total variation in the outcome that can be attributed to a specific predictor. Eta^2^ levels around 0.01 were considered to be small, around 0.10 as medium and around 0.25 as large [26]. In these models, the interaction effect of gender in the relation between sedentary behaviour, physical activity as well as dairy consumption and BMC/aBMD was tested by including an interaction factor. To calculate this interaction factor, gender was coded ‘+1’ for girls and ‘-1’ for boys and multiplied with the dairy consumption, sedentary behaviour or one of the different physical activity parameters after standardisation. If interaction was found in a model, this model was re-run after stratification for gender.

In a second set of regression analyses, we included both a parameter of sedentary behaviour or physical activity as well as the dairy consumption and looked at the associations with BMC and aBMD. First, these models were run including interaction factors to test the interaction effect between dairy consumption and sedentary behaviour as well as physical activity on BMC and aBMD. To calculate these interaction factors, the predictors were first standardised and then multiplied with each other. When an interaction factor showed a significant association with the dependent variable, plots of interactions were created where each factor variable was split into two groups (below and above the median value) to reflect low and high levels of the factors and to ensure an even distribution of the data. The interaction plots were created using the statistical software R3.1.0 (http://www.R-project.org).

## Results

### Descriptive statistics

Characteristics of the whole group, as well as for boys and girls separately, are shown in Table 1. Of the 272 participants, only 210 children had matching data from accelerometry. There was no significant difference in age (p = 0.551) and PEL (p = 0.900) between the group with and without accelerometer data. In the subgroup without accelerometer data, the proportion of children in which pubertal development had started was smaller (24 %) compared to the subgroup having accelerometer data (46 %); however the difference was not significant (p = 0.068).

**Table 1:** Subject characteristics for the whole study group and for boys and girls separately, as well as the p-value for the comparison between boys and girls

	Whole group		Boys		Girls		Boys versus girls
	N	Mean (SD)	N	Mean (SD)	N	Mean (SD)	p-value
**Socio**-**demographic**							
Age (years)	272	9.8 (1.4)	133	9.8 (1.5)	139	9.8 (1.4)	0.86^a^
Height (m)	272	1.4 (0.1)	133	1.4 (0.1)	139	1.4 (0.1)	0.81^a^
Tanner stage	272		133		139		0.22^b^
No signs of puberty (%)		71		74		68	
Signs of puberty (%)		29		26		32	
PEL Level (≤3/4/5or 6) (%)	263	32.7/14.8/52.5	128	28.1/14.4/57.8	135	35.0/15.6/47.4	0.22 ^b^
**DXA**: **Whole body minus head**	272		133		139		
BMC (g)		777 (177)		770 (165.5)		784 (188)	0.79^c^
BMC z-score		−0.20 (0.91)		−0.19 (0.91)		−0.20 (0.92)	0.93^a^
Areal BMD (g/cm^2^)		0.68 (0.08)		0.68 (0.07)		0.68 (0.08)	0.73^a^
Areal BMD z-score		−0.17 (0.86)		−0.16 (0.83)		−0.18 (0.89)	0.84^a^
**DXA**: **Whole body**	272		133		139		
Fat mass (g)		8540 (3646)		7313 (2816)		9717 (3625)	<0.01^c^
FMI (kg/m^2^)		4.27 (1.54)		3.65 (1.16)		4.86 (1.62)	<0.01^c^
Lean mass (g)		24169 (4750)		24522 (4401)		23832 (5055)	0.23^a^
FFMI (kg/m^2^)		12.09 (1.08)		12.31 (0.96)		11.88 (1.15)	<0.01^c^
**Nutrition**	264		130		134		
Dairy consumption (g/week)		1953 (1159)		2048 (1122)		1861 (1190)	0.19^a^
**Accelerometry**	210		105		105		
Sedentary time (%)^d^		57.7 (7.3)		56.9 (7.6)		58.8 (6.9)	0.09^a^
Light PA (%)^d^		34.6 (5.5)		34.5 (5.7)		34.5 (5.2)	0.82^a^
Moderate PA (%)^d^		5.0 (1.9)		5.7 (2)		4.4 (1.5)	<0.01^a^
Vigorous PA (%)^d^		2.7 (1.8)		2.9 (1.8)		2.4 (1.7)	0.01^c^
MVPA (%)^d^		7.7 (3.3)		8.7 (3.5)		6.7 (2.9)	<0.01^a^

Abbreviations: PEL, Parental education level; DXA, Dual-energy X-ray Absorptiometry; BMC, Bone Mineral Content; BMD, Bone Mineral Density; FMI, fat mass index; FFMI, fat-free mass index; PA, Physical Activity; MVPA, Moderate to Vigorous Physical Activity;^a^T-test;^b^Chi-square test; ^c^Mann-Whitney U-test; ^d^% of recorded time

Boys did not differ significantly from girls in age, pubertal stage, PEL level, BMC (z-score), aBMD (z-score), sedentary behaviour and lean mass. Physical activity (moderate, vigorous activity, MVPA) and FFMI were significantly higher in boys compared to girls and fat mass and FMI were significantly higher in girls compared to boys. No significant differences in aBMD and BMC were found between PEL groups (analyses not shown).

### Associations between sedentary behaviour, physical activity, dairy consumption and bone parameters

Results of the regression analyses studying the association of sedentary behaviour, physical activity and dairy consumption with BMC (z-score) and aBMD (z-score) after adjusting for age, gender, Tanner stage and body composition are presented in Table 2.

**Table 2:** Standardised regression coefficients, p-value and eta^2^ for the association of physical activity and dairy consumption with BMC (g) and areal BMD (g/cm^2^) as well as BMC z-score and BMD z-score of the whole body minus head

	BMC (g)^b^			areal BMD (g/cm^2^)^b^			BMC z-score^c^			BMD z-score^c^		
	β	p	eta^2^	β	p	eta^2^	β	p	eta^2^	β	p	eta^2^
Sedentary time (%)^a^	−0.021	0.462	0.0004	−0.040	0.236	0.0020	−0.113	0.092	0.0103	**−0.156**	**0.018**	0.0143
Light PA (%)^a^	0.031	0.252	0.0009	0.320	0.331	0.0010	**0.134**	**0.040**	0.0125	**0.148**	**0.023**	0.0078
Moderate PA (%)^a^	−0.006	0.838	0.0000	0.018	0.607	0.0002	−0.002	0.981	0.0006	0.061	0.381	0.0014
Vigorous PA (%)^a^	−0.008	0.752	0.0001	0.041	0.196	0.0020	0.038	0.571	0.0055	0.111	0.095	0.0275
MVPA (%)^a^	−0.009	0.755	0.0001	0.033	0.328	0.0010	0.019	0.783	0.0008	0.097	0.159	0.0135
Dairy consumption (g/week)	**0.078**	**0.001**	0.0068	**0.079**	**0.003**	0.0072	**0.173**	**0.002**	0.1070	**0.120**	**0.029**	0.0497

Significant association are indicated in bold; Abbreviations: β, standardised regression coefficient; BMC, Bone Mineral Content; BMD, Bone Mineral Density; FMI, fat mass index; FFMI, fat-free mass index; PA, Physical Activity; MVPA, Moderate to Vigorous Physical Activity; η^2^, the proportion of the total variance that is explained by the predictor; ^a^ % of recorded time; ^b^regression model adjusted for age, gender, Tanner stage, height, FMI and FFMI; ^c^regression model adjusted for Tanner stage, FMI and FFMI

**Fig. 1:**
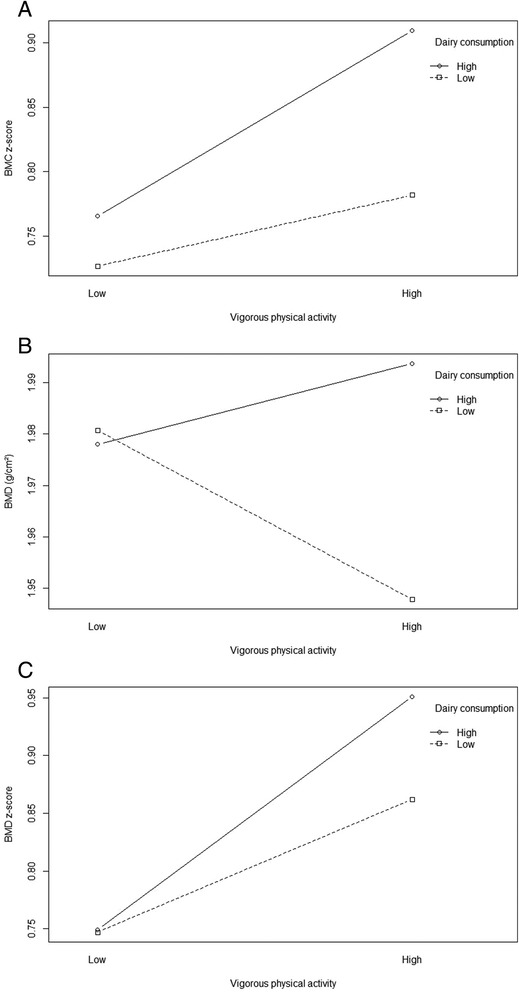
Plots of interactions identified in the regression models for diary consumption and vigorous physical activity interaction on **a**) BMC z-score, **b**) aBMD and **c**) aBMD z-score. The curves represent the outcome parameters’ geometric mean at different levels of dairy consumption and vigorous activity

Dairy consumption showed a significant and positive association with BMC and aBMD (both the absolute values and z-scores) (Table 2). The eta^2^ of dairy consumption in the models with absolute values of BMC and aBMD were very low, whereas a small to medium eta^2^ was found for dairy consumption in the models using z-scores of BMC and aBMD. Time spent in sedentary behaviour showed a significant and negative association with aBMD z-score although the eta^2^ value was small. Time spent in light physical activity was significantly associated with BMC z-score and aBMD z-score. For the other physical activity parameters, no significant associations with BMC (z-score) nor aBMD (z-score) was found (Table 2). Moreover, gender interactions were tested, but not present. Next, linear regression models were run to test whether interaction effects were present between dairy consumption and the parameters of sedentary behaviour or physical activity. A significant interaction effect between dairy consumption and vigorous physical activity (VPA) was found on BMC z-score as well as aBMD (absolute value as well as z-score). Interaction plots are shown in Fig. 1, indicating that children having both high dairy consumption and high VPA (above the median level) have the highest values for BMC z-score and aBMD (z-score).

## Discussion

This study indicated that in 6 to 12 years old children dairy consumption was positively associated with BMC and aBMD (absolute value as well as z-score) of the whole body minus the head. Next, SB was negatively associated with BMD z-score and light PA was positively associated with both BMC and aBMD z-score. Moreover, an interaction effect between VPA and dairy consumption on BMD (z-score) and BMC z-score was found, indicating that children with both high VPA and high dairy consumption had higher values of whole body BMC and aBMD. No interaction effects with gender were found.

### Sedentary behaviour and physical activity

It can be concluded from this investigation that sedentary behaviour is negatively related to bone parameters and that VPA is positively related to bone parameters, in the presence of medium to high dairy consumption. A previous study, using DXA and accelerometers, showed similar results concerning the positive effect of physical activity in 11 year old boys and girls [27]. Also a 3-year follow-up study of children with a median baseline age of 5.3 years, using also DXA and accelerometers, indicated that physical activity contributed to the increase in BMC [28].

Considering the association between bone parameters and sedentary behaviour, this study is one of the first studies indicating the negative association between objectively measured sedentary behaviour and BMC and aBMD of the total body minus the head in young children. Very recently, an American study was published based on data of a cohort of children between 8 and 22 years old, indicating that time spent in screen-based sedentary behaviours is negatively associated with femoral BMC and spinal BMC [15]. Similar associations were already described by Gracia-Marco et al. [13] in Spanish adolescents, using questionnaire data on sedentary behaviour. They found a negative association between the time spent studying and whole body BMC in girls; however, this association was no longer significant after adjustment for lean mass. In Spanish adolescent boys, total sedentary behaviour was negatively associated with whole body BMC, but significance disappeared after adjustment for lean mass. In addition, the use of internet for non-study was negatively associated with whole body BMC after adjustment for lean mass [13]. These results are in line with another Spanish study also using an adolescent study population reporting a negative association between hours spent watching TV and BMC, but only in boys [14]. Moreover, Vatueña et al. [29] indicated that physical activity interacts with vitamin D status in adolescents, resulting in an improvement of the bone mass when being physically active and vitamin D sufficient. However, no positive effect of a sufficient vitamin D status on bone was seen in physically inactive adolescents.

### Dairy consumption

Dairy consumption was positively associated with BMC and aBMD (absolute value and z-score) in our study. Most studies focus on the effect of calcium intake, however, we decided to assess the influence of dairy consumption in general as dairy products are considered as an optimal source of calcium as well as other limiting nutrients (potassium, magnesium, zinc and phosphorus), with important effects on bone health. Dairy products may represent the best sources of calcium-containing food due to their high content, high absorptive rate and relatively low cost [30].

Previous cross-sectional studies investigating the role of dairy consumption and/or calcium have shown conflicting results: while some studies found a positive association between calcium intake and BMC/aBMD [31, 32], in others there was no relation between bone mass and calcium intake during childhood [9, 33, 34]. This contradiction could be explained by the deficit of standardization of food measurement methods. When studying the effect of dairy and/or calcium on bone mineral development, lifetime dairy and/or calcium intake may be a more important factor than calcium intake assessed on a single day or short term period [32]. For this reason, the use of our FFQ can be considered as a strength because it aimed to assess the usual consumption, instead of the consumption on a single time point. Although necessary, adequate calcium intake may not be sufficient in itself for optimal bone development [35].

### Interaction dairy consumption and physical activity

We observed an interaction effect between dairy consumption and VPA on BMC z-score and aBMD (absolute values and z-score) of the whole body minus the head. This is in line with some other studies showing a significant interaction among physical activity and calcium intake in prepubertal (8-11 years) children [32, 33, 36]. In some of these studies the interaction was only observed when VPA was considered, but not when physical activity was classified as total physical activity [32, 36], which is in line with our results. These results are confirm existing evidence that BMC is higher only if both calcium and VPA are high, while no such benefits are obtained from high levels of only one variable [36] and hence that they do not act on bone independently from each other. Calcium seems necessary for exercise to have an optimal bone-stimulating action [3, 37]. However, considering adequate nutrient intakes for optimising bone geometry and strength, other authors concluded that exercise appears to be more important than nutrient intakes and that the role of exercise is not controlled by nutritional factors [11].

### Strengths and limitations

A first strength of our study is the use of an accelerometer in assessing physical activity. This method provides a more precise and direct quantification of physical activity than self-reported questionnaires. Because uniaxial accelerometers detect motion in vertical plane, which is likely to reflect weight-bearing activity, this technique may be particularly useful in evaluating the relationship between physical activity and skeletal development in childhood [27]. In addition, short intervals (15 s epochs) were used, while most other studies used a 1-min time interval during recording [4, 10, 11] . Therefore, it is less likely that our accelerometry movement count data missed shorter periods of high-intensity physical activity. Different types of accelerometers were used in this study. However, Robusto et al. [38] recently indicated that there is strong agreement between the GT1M, GT3X, and GT3X+ activity monitors, thus making it acceptable to use different ActiGraph™ models within a given study. Unfortunately we have no information on the different kinds of activities the children performed. In addition, sports like swimming were not measured. Since water sports are not weight-bearing sports, we think that the effect of not considering this kind of activities is limited [39, 40]. Secondly, we were able to study the daily activities of a healthy group of school children [41]. Our findings complete the evidence for the osteogenic effect of physical activity on bone development from either observational studies of athletes or targeted intervention studies [28, 42]. Thirdly, the adjustment for potential confounders such as age, gender, Tanner stage and body composition also strengthens our study. Indeed, sexual maturation has an influence on physical activity levels and the skeleton is more responsive to mechanical load in the late pre- or early peripubertal period [43, 44]. Finally, sedentary behaviour, physical activity as well as dairy consumption were measured in this study while different previous studies included either only physical activity or only dietary information.

Limitations of our research include its cross-sectional design, making it impossible to exclude the possibility that relationships were caused by reverse causation or unmeasured confounders. A second limitation is the inability of the DXA measurement technique to capture bone volume. Consequently, the same increase in aBMD could be the result of increased bone mineral or of increased bone size. Despite this limitation, DXA is the most commonly used densitometric method for assessing bone health in adults and children, due to its low radiation exposure, its high precision and accuracy and predictive value. In contrast to pQCT and QUS, information of the whole body can be obtained [45]. Third, children were not selected randomly but were recruited in the framework of an on-going study focussing on children living in one commune. Based on the PEL data available, it is know that the average socio-economic status of the study sample is quite high. Therefore, the sample may not be considered to be representative for all Flemish children. To take this into account, we checked whether the bone health of the children was influenced by the PEL level, however it was not the case. This confirms earlier findings that there is no link between socio-economic status and bone mass in children [46]. Fourth, dairy consumption was measured with a FFQ, which is a method containing a substantial amount of measurement error. The details of dietary intake collected with this method are limited and the quantification of intake is not as accurate as with 24 h recalls or dietary records. Nevertheless, the FFQ used in this study was validated and demonstrated good repeatability and fairly good ability to classify subjects into extremes of Ca intake [24]. Also in other studies, a FFQ was used to assess calcium intake in children, e.g. in the Amsterdam Growth and Health Longitudinal Study in adolescents [47].

## Conclusion

In conclusion, we found that in 6 to 12 years old children dairy consumption was positively associated with bone parameters of the whole body minus the head. Next, SB was negatively associated and light PA was positively associated with bone parameters. Moreover, an interaction effect between VPA and dairy consumption on bone parameters was found, indicating that children with both high VPA and high dairy consumption had higher values of whole body BMC and aBMD. As a result, we conclude that promoting physical activity and dairy consumption and decreasing sedentary behaviour in young children will help to maximize bone mass, which can help in the prevention of osteoporosis later in life.
